# In-Situ Simulation Enhances Emergency Preparedness in Pediatric Care Practices

**DOI:** 10.7759/cureus.3389

**Published:** 2018-10-01

**Authors:** Shiva Kalidindi, Michael Kirk, Elliot Griffith

**Affiliations:** 1 Emergency Medicine, University of Central Florida College of Medicine, Orlando, USA; 2 Miscellanous, University of Central Florida College of Medicine, Orlando, USA; 3 Internal Medicine, University of Central Florida College of Medicine, Orlando, USA

**Keywords:** primary care, emergency medicine, pediatrics, simulation, training, in-situ, pediatric emergency medicine, urgent care

## Abstract

Background

It is not uncommon for emergencies to present at primary care offices. As such, it is necessary for those offices to be prepared to handle, at a minimum, the most common types of emergencies.

Objective

To evaluate the effectiveness of in-situ simulation training in improving emergency preparedness within pediatric primary care settings.

Methods

Simulation training was provided at 20 primary care offices in Central Florida. The participants were asked to complete a pre-simulation survey that utilized a five-point Likert-type scale to evaluate office preparedness and the confidence of staff members in managing emergency presentations within their settings. Subsequent to the simulation, participants were asked to complete a post-survey to evaluate the effectiveness of the simulation training.

Results

Primary care office staff members reported an enhanced preparedness in managing emergencies post-simulation training (pre-simulation 2.95 vs. post-simulation 4.02; p-value<0.05). They also reported higher levels of comfort in managing emergency situations after the simulation training (pre-simulation 3.22 vs. post-simulation 4.53; p-value<0.05). Overall, 100% of participants found the simulation to be effective or extremely effective.

Conclusions

Our data suggests that the simulation training has improved office preparedness in managing emergencies in a pediatric primary care setting. The simulation training has also been shown to improve the comfort level of pediatric primary care office staff in handling emergency situations. This study was limited to pediatric primary care settings in the Central Florida region, and it is unclear if the findings of this study are generalizable to all primary care practices. Further studies are required to explore whether such training can result in practice change and improve outcomes for more patients.

## Introduction

Hospitals and acute care settings expect to take care of patients presenting with emergent conditions. Emergencies do occur in the primary care setting, although infrequently. However, primary care offices are often not prepared to handle them. In one study, 62% of primary care physicians, including both pediatricians and family physicians in an urban setting, report having one patient each week that typically requires emergent stabilization or further hospital treatment [1]. Another study which surveyed 52 pediatric offices found that these offices were seeing two emergencies per month [2]. It is clear that even though most emergencies present to hospitals, they are not uncommon in the primary care setting, and this requires that primary care offices be prepared to manage these presentations until the arrival of emergency medical services.

The most common types of medical emergencies in primary care offices, including both pediatric and family medicine, are asthma exacerbations and other respiratory emergencies, chest pain, anaphylaxis, hypoglycemia, seizures, impaired consciousness, dehydration, and infections in young infants [3-4]. With many patients opting to go to a primary care physician whom they know and trust, the office serves as the frontline of emergency care where vital and lifesaving care may be required to be administered. The best way to be prepared for such emergencies is to practice on a regular basis in the office setting, with as much of the staff as possible. Simulations are excellent instruments for practising skills and emergencies before having to use them on an actual patient. Office staff may only have one chance to provide care for someone with a life-threatening condition, so it is important to be prepared. Having a regular simulation program will reduce anxiety when facing urgent issues, ensures that medication and equipment are current and readily available, and identifies any type of problem that may need to be addressed [4-5].

Primary care offices typically do not have as much training in emergent situations and, therefore, many physicians and staff are not adequately prepared. Due to the lower volume of emergencies, the typical and older “see one, do one, teach one” model of learning is no longer practical or sufficient for office preparedness [6]. Not only can knowledge-based skills be out of practice, but other concerns may also be present. In order to adequately operate in an emergent situation, there needs to be a clear role for every staff member for effective communication and teamwork. Problems can also occur in the emergency supplies and equipment. Even though certain things may be “standardized,” they often vary from clinic to clinic [7]. These variations and the lack of familiarity can delay response times that may adversely affect patient outcomes.

Simulation-based training has long been used in critical care, emergency departments, and medical transport to improve preparedness and responsiveness to emergent situations [7]. The goals of simulations are not only to improve the participants' knowledge-based skills but also to recognize operational deficiencies, optimize processes, familiarize participants with available equipment, improve team dynamics, and create effective communication. Studies have shown that simulation improves learning, provides the opportunity to learn new procedures, refines skills, boosts confidence, and most importantly, improves clinical outcomes [5,8]. These studies have shown that hospital staffs can improve with simulation training, but there is little data on how simulation can improve emergency response in a primary care setting.

Thousands of new healthcare providers graduate and obtain licensure each year in the United States, and as other studies have noted, federal agencies and academics alike have deemed training in emergency preparedness is vital [9-10]. This study has evaluated the effectiveness of in-situ simulation experiences in enhancing the emergency preparedness among pediatric primary care practices. We hypothesized that “in-situ simulation improves emergency preparedness of pediatric care practices”. To investigate this hypothesis, this study assessed the current preparedness of those pediatric primary care practices. We believe that the training required to improve emergency preparedness in primary care offices can be obtained via simulation.

## Materials and methods

Study participants

Twenty pediatric primary care practices affiliated with Nemours Children’s Hospital were invited to take part in the study voluntarily. These practices encompass a wide demographic and are representative of urban, suburban, and rural areas in the region. All medical and nonmedical staff in each of the participating practices were requested to take part in the study. For the purposes of the survey, the clinic team was separated into two categories: Group 1 was comprised of providers; Group 2 was comprised of physician assistants (PA), registered nurses (RN), medical assistants (MA), and all other staff. This was simply to get an idea if there were different opinions on how the exercise benefitted the practice overall. There may be variances between physicians and staff members in the quality of care provided, based on how well the team communicated. During the exercise, both groups of participants worked together to accomplish the set goal.

Pre-simulation self-assessment

At the beginning of each office’s participation, all of the active staff were asked to complete a self-assessment survey intended to evaluate baseline participant skills, comfort level in emergency presentations, and their preparedness. This allowed for post-survey data to be compared to see if the simulation made a difference in the staff's confidence in certain skills and/or techniques. The questions provided to both groups of participants varied slightly as the physician group contained more questions (Table 1).

**Table 1:** Survey questions provided to participants

Survey questions for support staff	Survey questions for physicians
How would you rate the current preparedness in your office for handling emergent presentations?	How would you rate the current preparedness in your office for handling emergent presentations?
Rate your comfort level in the following skills: cardiopulmonary resuscitation (CPR), automated external defibrillator (AED) administration, and intramuscular (IM) epinephrine administration	Rate your comfort level in the following skills: cardiopulmonary resuscitation (CPR), automated external defibrillator (AED) administration, and intramuscular (IM) epinephrine administration
Rate your comfort level in handling the following emergency situations: asthma exacerbation, anaphylaxis, seizures, cardiac arrest	Rate your comfort level in handling the following emergency situations: asthma exacerbation, anaphylaxis, seizures, cardiac arrest
What is your prior experience with simulation-based learning?	What is your prior experience with simulation-based learning?
	Rate the following processes in case of an office emergency: availability of equipment, staff’s awareness of equipment/supplies, and clear roles are defined during an emergency
	How would you rate communication among staff in case of an emergency situation?
	How often has your team practiced responding to emergency situations at your office location?

The responses were based on a simple five-point ranking system. The responses vary based on a reasonable answer to the specific question, but each response will have a five-point ranking system. For example, the question, “What is your prior experience with simulation-based learning?” will have the responses: none, very little, some, more than average, and frequent. Whereas the question, “What is your current comfort level in handling asthma exacerbation?” will have the possible responses: Not comfortable, somewhat comfortable, comfortable, very comfortable, or extremely comfortable.

Simulation

The primary care office staff members and providers were oriented to the simulation manikin by a simulation specialist. Once oriented to the manikin, the staff participated in an in-situ simulation experience emulating emergent pediatric presentations in their practice. These situations included an asthma-exacerbated child, a child in cardiac arrest during an episode of septic shock, a seizing child, and a child with a severe allergic reaction. Subsequent to this experience, participants were provided a chance to reflect on their experiences, share their thoughts, and to identify opportunities for improvement. Following the reflection, the participants were provided with another opportunity to incorporate identified changes into practices and processes by engaging in an identical simulation experience. This hopefully solidified certain aspects of the case that may benefit the office in the future. Those aspects included, but were not limited to, defining clear roles during an emergency, providing an emergency guideline that is available to the entire team, or going over certain skills or techniques that may have been forgotten (i.e., cardiopulmonary resuscitation (CPR), epinephrine administration, use of the automated external defibrillator (AED), etc.).

Post-simulation self-assessment

The practice teams were asked to complete a survey that evaluated their emergency preparedness and the effectiveness of the simulation experience. The questions were identical to the questions from the pre-survey. This allowed for a direct comparison to how the simulation affected the healthcare team’s confidence in certain areas pertaining to specific emergent situations. This was followed up by another survey six months after the simulation experience. The purpose of the follow-up survey was to evaluate if the simulation experience had resulted in a practice change and if these changes have had an impact on patient outcomes. This survey included similar questions as well as an open response which allowed feedback as to whether or not the simulation experience had had an effect in any situation that the clinic may have had to deal with within six months.

Statistical analysis

With continuous data that was collected from multiple independent groups, the results are best visualized as a mean and standard deviation with 95% confidence intervals. This is beneficial in analyzing whether or not the simulation had had any benefit in regard to the office staff’s confidence in their skills and preparedness in their abilities during an emergency situation. Independent factors such as the level of participant confidence in each category were compared to each other and were used to determine if there was an aspect of simulation training that had had more of a benefit than other categories.

## Results

Primary care office staff members reported enhanced preparedness in managing emergencies post-simulation training (pre-simulation 2.95 vs. post-simulation 4.02). They also reported higher levels of confidence in managing emergency situations after the simulation training (pre-simulation 3.22 vs. post-simulation 4.53). These were significant improvements with a p-value <0.05. Primary care providers did not report such enhanced preparedness in managing emergencies following the simulation-based training (pre-simulation 3.89 vs. post-simulation 2.89). Overall, 100% of participants found the simulation was at least effective.

The six-month follow up surveys from the clinics that were visited reported a retention of confidence in office preparedness and managing emergencies. It was also reported that staff could easily locate tools in their office to provide oxygen to a patient receiving breathing treatments as a result of the post-simulation discussion.

## Discussion

In order for simulation-based training to be effective, there are certain aspects that need to be examined. Simulation is valuable only if it closely mimics a true clinical situation or patient encounter and is practiced on a regular basis. It is important to involve the entire staff if possible, with clearly determined roles. All the members of the staff should be well-versed in their role if an emergency event were to occur to ensure safe and effective treatment. The simulation should be treated as a real patient encounter by physically performing all tasks that would normally be completed. In other words, do as one would normally do in a real-life situation. This includes actually handling the equipment and drawing up simulation medications. At the end of the simulation, it is important to critique and reflect on the exercise. Finding out what was done well and what could be improved should be identified. Through these are only reflections, office staff can truly become a team when an emergency arises. It is important to understand that even the most well-run primary care practice can benefit from a careful and constructive examination of their supplies, medications, and protocols [7].

**Figure 1:**
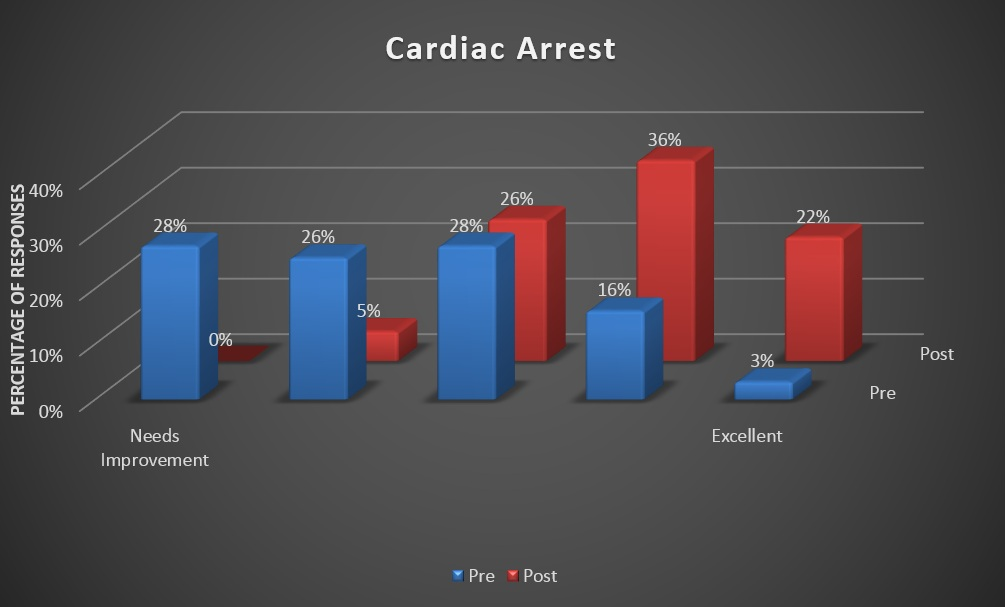
Staff confidence in handling a cardiac arrest presentation

**Figure 2:**
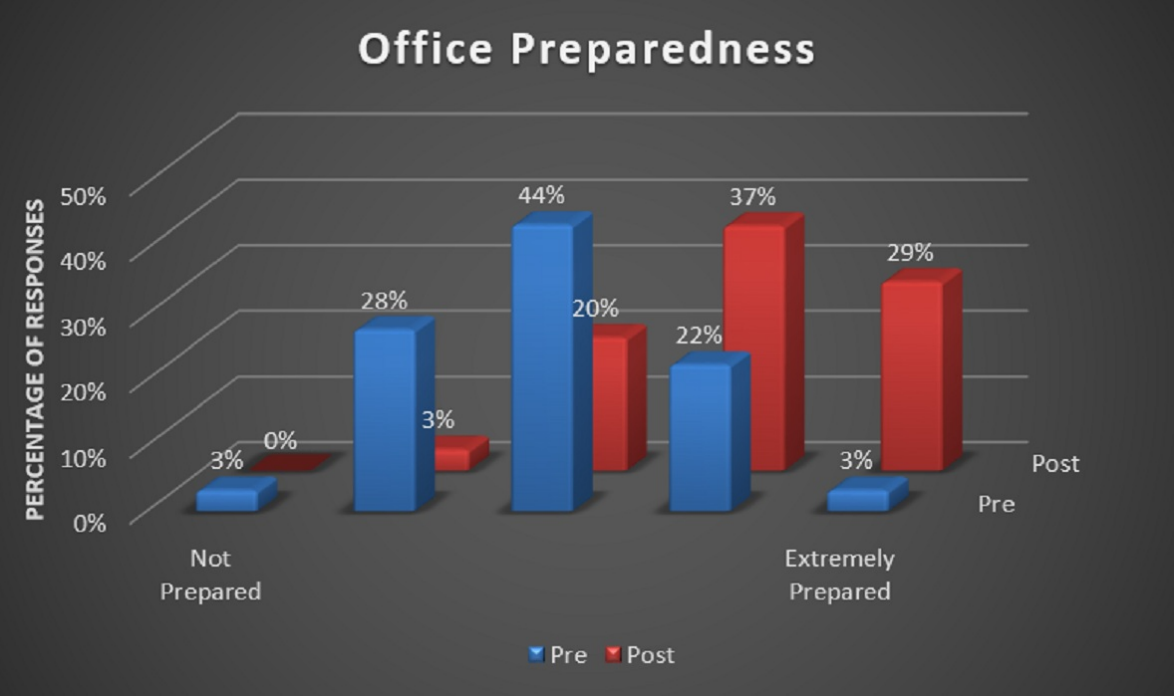
Staff confidence in office preparedness

**Figure 3:**
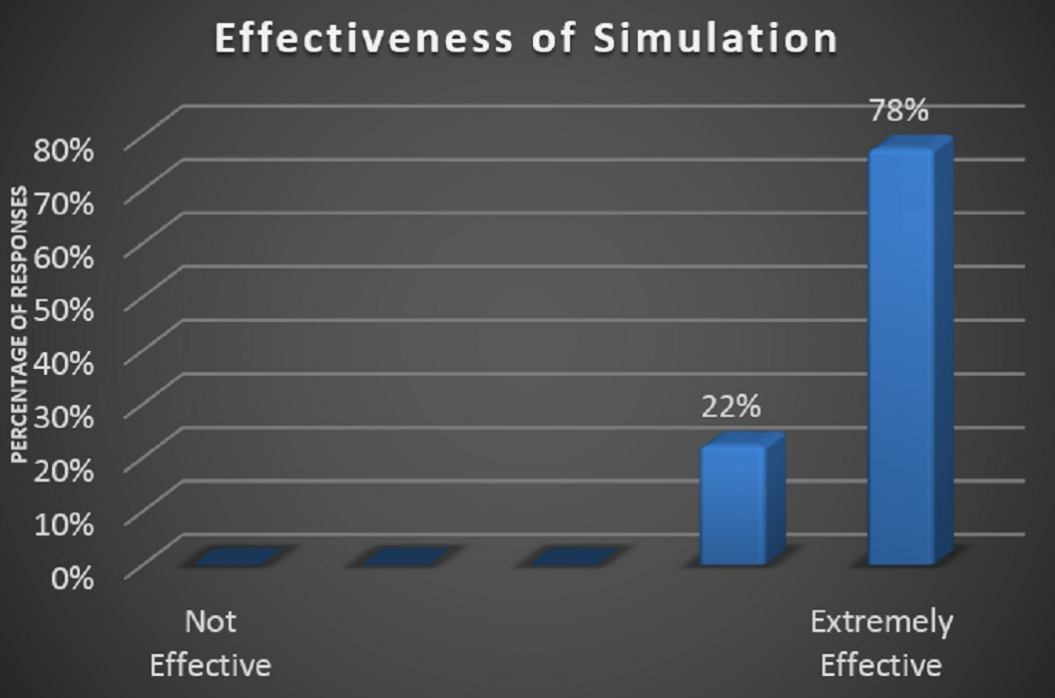
Overall perception of simulation effectiveness

During this experiment, we found that 100% of the pediatric primary care offices supporting staff members reported improvements in all the questions of the survey. Staff members reported the greatest benefit in handling cardiac arrest cases, which is demonstrated in Figure 1. The response for the current office preparedness proved to be most improved amongst the staff (Figure 2). There was also a 100% response about the simulation being effective or extremely effective by all participants (Figure 3). 

The inconsistencies in the perceived benefit of the training beween staff members and providers may be a result of over-confidence during pre-simulation. Even though the primary care offices did not see all the different types of simulations, they were still asked about them on the pre-survey and post-survey. It is possible that the providers may have been more confident in their staff members and their own abilities before experiencing a breakdown in communication, not having defined roles for everyone, not having proper protocols or guidelines in place, or their own lack of skills with lifesaving tools. A great source of selection bias in these types of studies is that the participants are simply volunteers. These are usually practices that may possibly believe that they could benefit from these exercises. The study may not include primary care practices that are already confident in their preparedness and therefore may overrepresent underprepared primary care practices. Future studies may consider stratifying practices that are based on their pre-simulation preparedness and confidence, in order to reduce these biases. 

## Conclusions

Our data suggests that simulation-based training improves office preparedness in managing emergencies in pediatric primary care settings. The experiment demonstrated that simulation-based training can expose weaknesses in primary care settings' ability to handle emergencies. If possible, in-situ simulation-based training should be considered in all primary care practices to ensure that the providers and staff members can handle at the least the most common presenting emergencies. The safest setting to experience those breakdowns in communication and roles are during simulation-based training, before a life is hanging in the balance.

While our study was confined to Nemours Children’s Hospital affiliated primary care clinics in Central Florida, we are confident that the results of this study are generalizable to other practice types. Further studies are required to explore whether such training can result in practice change and improve outcomes in patients. It should be noted that the accuracy of the results could be improved with the use of surveys specific to the simulation-based training that was completed at the time. This would reduce the change in confidence between pre-survey and post-survey for skills that were not a part of the training session. It would also have made it possible to determine which skills benefitted more than others by using simulation-based training and could have provided feedback to our team to determine areas of our simulation that could improve in order to benefit these primary care offices even more.

## References

[1] Fuchs S, Jaffe DM, Christoffel KK (1989). Pediatric emergencies in office practices: prevalence and office preparedness. Pediatrics.

[2] Flores G, Weinstock DJ (1996). The preparedness of pediatricians for emergencies in the office: what is broken, should we care, and how can we fix it?. Arch Pediatr Adolesc Med.

[3] Minkovitz CS, O’Connor KG, Grason H, Chandra A, Aligne CA, Kogan MD, Tayloe D (2008). Pediatricians' involvement in community child health from 1989 to 2004. Arch Pediatr Adolesc Med.

[4] Rothkopf L, Wirshup M (2013). A practical guide to emergency preparedness for office-based family physicians. Fam Pract Manag.

[5] Al-Elq AH (2010). Simulation-based medical teaching and learning. J Family Community Med.

[6] Vozenilek J, Huff JS, Reznek M, Gordon JA (2014). See one, do one, teach one: advanced technology in medical education. Acad Emerg Med.

[7] LaVelle BA, McLaughlin JJ (2008). Simulation-based education improves patient safety in ambulatory care. Advances in Patient Safety: New Directions and Alternative Approaches.

[8] Long RE (2005). Using simulation to teach resuscitation: an important patient safety tool. Crit Care Nurs Clin North Am.

[9] Williams J, Nocera M, Casteel C (2008). The effectiveness of disaster training for health care workers: a systematic review. Ann Emerg Med.

[10] Morrison AM, Catanzaro AM (2010). High-fidelity simulation and emergency preparedness. Public Health Nurs.

